# Structural basis of katanin p60:p80 complex formation

**DOI:** 10.1038/s41598-017-14194-2

**Published:** 2017-11-02

**Authors:** Lenka Rezabkova, Kai Jiang, Guido Capitani, Andrea E. Prota, Anna Akhmanova, Michel O. Steinmetz, Richard A. Kammerer

**Affiliations:** 1 Laboratory of Biomolecular Research, Division of Biology and Chemistry, Paul Scherrer Institut, CH-5232 Villigen PSI, Switzerland; 2 Cell Biology, Department of Biology, Faculty of Science, Utrecht University, Padualaan 8, 3584 CH Utrecht, The Netherlands; 3 Biozentrum, University of Basel, Basel, CH-4056 Switzerland

## Abstract

Interactions between microtubule (MT) interacting and trafficking (MIT) domains and their binding proteins are important for the accurate progression of many cellular processes that require the AAA+ ATPase machinery. Therefore, knowledge on the structural basis of MIT domain interactions is crucial for understanding the molecular mechanisms underlying AAA+ ATPase function. Katanin is a MT-severing AAA+ ATPase that consists of p60 and p80 subunits. Although, the hexameric p60 subunit is active alone, its association with the p80 subunit greatly enhances both the MT-binding and -severing activities of katanin. However, the molecular mechanism of how the p80 subunit contributes to katanin function is currently unknown. Here, we structurally and functionally characterized the interaction between the two katanin subunits that is mediated by the p60-MIT domain and the p80 C-terminal domain (p80-CTD). We show that p60-MIT and p80-CTD form a tight heterodimeric complex, whose high-resolution structure we determined by X-ray crystallography. Based on the crystal structure, we identified two conserved charged residues that are important for p60-MIT:p80-CTD complex formation and katanin function. Moreover, p60-MIT was compared with other MIT domain structures and similarities are discussed.

## Introduction

Katanin, spastin and fidgetin are closely related MT-severing enzymes that possess the unique property to catalyse the removal of tubulin heterodimers from the interior of a MT lattice, which results in the breakage of MTs into shorter fragments. The proteins play an essential role in a wide range of important cellular activities, including mitosis, meiosis, cilia disassembly, migration and neuronal morphogenesis, function and plasticity^1,2^. The enzymes belong to the meiotic clade subfamily of ATPases associated with diverse cellular activities (AAA+). The fourth member of this subfamily is vacuolar protein sorting 4 (VPS4) that promotes the disassembly of endosomal sorting complex required for transport III (ESCRTIII) proteins from the membrane. The hallmark of AAA+ proteins is the presence of a conserved AAA+ domain that contains the ATPase activity. Members of the AAA+ protein family generally function as hexameric or dodecameric stacked ring-shaped oligomers^3^.

Katanin is composed of two subunits, p60 and p80, which were named according to their molecular weight of 60 and 80 kDa, respectively. The regulatory p80 subunit contains an N-terminal WD40 domain that is responsible for targeting katanin to the centrosome, followed by a central proline-rich region and C-terminal domain (p80-CTD), which is required for hetero-dimerization with the p60 subunit. The catalytic p60 subunit comprises an N-terminal MT-interacting and -trafficking domain (p60-MIT) that interacts with p80-CTD and a C-terminal AAA+ domain that binds and hydrolyses ATP. ADP-bound p60 is typically monomeric and exchange of ADP to ATP induces hexamerization, a process which is necessary for the protein’s MT-severing activity^2,4^.

Katanin was shown to localize to spindle poles during mitosis in most animal cells and plays an important role in spindle organization^5–9^. It has been identified as a crucial regulator of early embryonic development and recessive mutations or deletions in the katanin-encoding genes are responsible for a dramatic increase in the number of centrosomes and multipolar spindles. These abnormal processes are thought to lead to severe microcephaly or microlissencephaly^5,6^, neurodevelopmental diseases that are characterized by a small brain and mental retardation. Moreover, katanin plays a critical role in plants where its function is required for the bending of the plant axis toward the light source^10^.

Although recent cell biology data emphasize the crucial role of katanin in severe diseases such as microcephaly, the structural mechanism underlying MT-binding and -severing by katanin is only poorly understood. The current model of MT severing is based on spastin data suggesting that the ring-shaped complexes formed by the AAA+ domains dock onto the MT lattice, which results in an interaction between the positively charged N-terminal pore entrance of the AAA+ ring and the negatively-charged C terminus of tubulin. The linker and the MIT domains extending from the ring make additional contacts with the MT lattice, thereby increasing the binding of spastin to MTs. Potentially, these additional interactions also stabilize the ATP-induced hexameric spastin structure on MTs. According to the proposed model, spastin then pulls the C terminus of tubulin through the central pore formed by the hexameric ring of AAA+ domains, thereby generating a mechanical force that destabilizes tubulin–tubulin interactions within the MT lattice and causes MT breakage^11^. As a result of the similar domain organization and high sequence identity between spastin and the p60 subunit of katanin, the proposed mechanism might be similar for katanin, which consists of two subunits, however. Although, the hexameric p60 subunit is active alone, the p80 subunit greatly enhances both the MT-binding and -severing activities of katanin. However, the molecular mechanism of how the p80 subunit contributes to katanin function remains to be elucidated.

Recently, we investigated the structure-function relationship of katanin complexed with abnormal spindle-like microcephaly-associated protein (ASPM)^12^. We revealed that the katanin:ASPM complex regulates MT dynamics at the spindle pole. We demonstrated by X-ray crystallography that conserved motifs in ASPM bind at the interface formed by the N- and C-terminal domains of the katanin p60 and p80 subunits, respectively. While in the previous work we limited our structural analysis mainly to the interaction between ASPM and katanin, in the present complementary study we focused on the molecular details of the heterodimeric p60-MIT:p80-CTD structure^12^. Towards this end, we biophysically characterized the p60-MIT:p80-CTD heterodimeric complex in solution and solved the high-resolution crystal structure of the p60-MIT:p80-CTD complex by X-ray crystallography. On the basis of this structure, we identified two conserved charged residues that are important for heterodimeric p60-MIT:p80-CTD complex formation and katanin function. Moreover, a comparison with other MIT domain structures was carried out.

## Results and Discussion

### Crystal structure of the heterodimeric p60-MIT:p80-CTD complex

**Figure 1:**
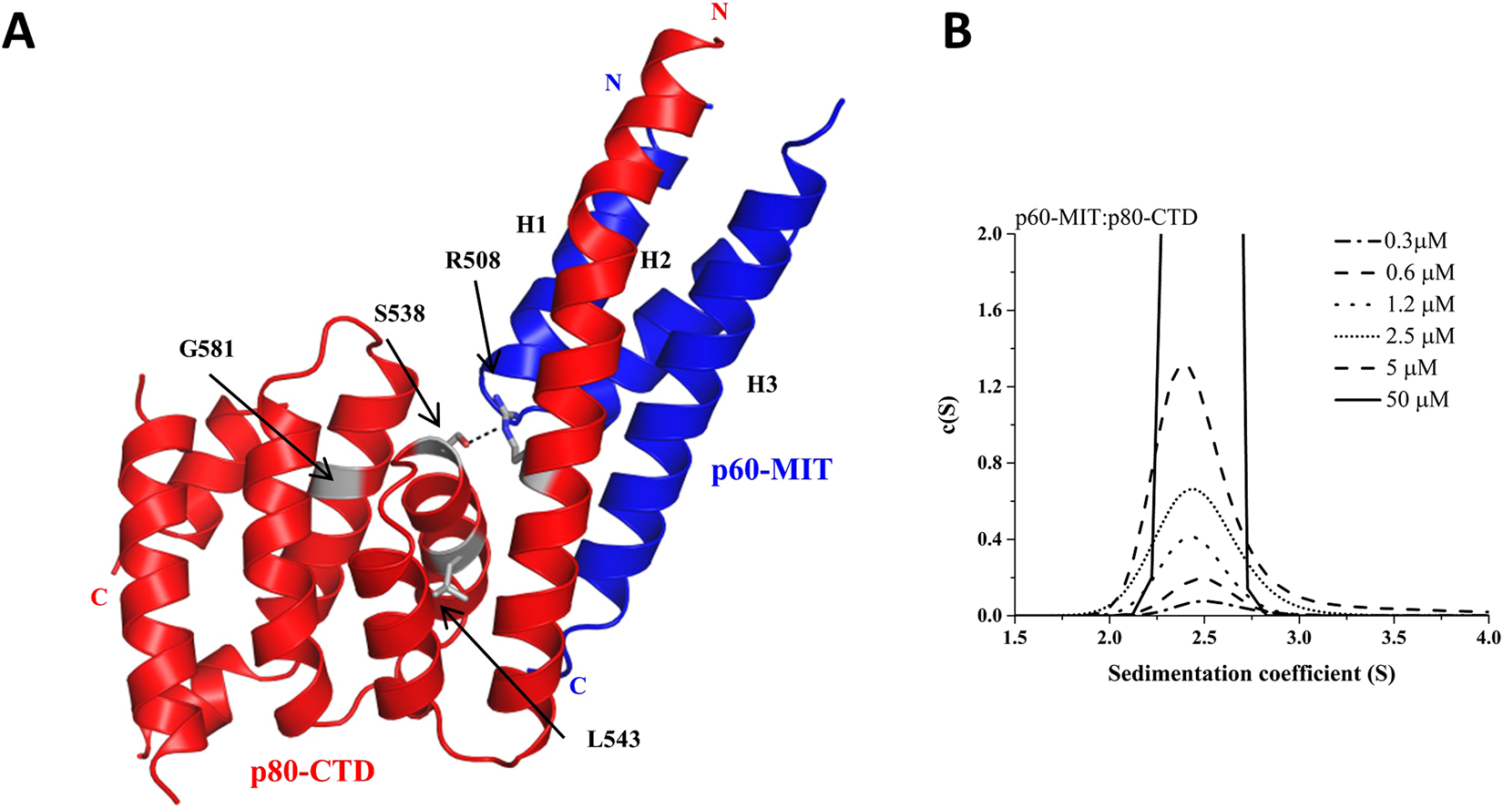
Biophysical and structural characterization of the p60-MIT:p80-CTD complex by AUC and X-ray crystallography. (**A**) Cartoon representation of the crystal structure of the p60-MIT L40P:p80-CTD complex. Blue, p60-MIT; red, p80-CTD. Microcephaly-associated mutations S538L, L543R and G581D, are highlighted as grey sticks. The intradomain hydrogen bond formed between S538 and R508 is indicated by a black dotted line. (**B**) c(S) distribution analysis of the p60-MIT:p80-CTD complex.

The N-terminal MIT domain of the p60 subunit (aa 1–78 of mouse p60) forms a complex with the C-terminal domain of the p80 subunit (aa 481–658 of mouse p80), an interaction that greatly enhances both the MT-binding and -severing activities of katanin^13^. To understand at the molecular level how p60-MIT recognizes p80-CTD, we determined the X-ray crystal structure of the heterodimeric p60-MIT:p80-CTD complex (Fig. 1A). We did not manage to obtain well-diffracting crystals for the wild-type complex; however, crystals suitable for structure determination were obtained with a p60-MIT mutant in which L40 was substituted by P. This residue was probably introduced by chance during the cloning process of the katanin subunits because no sequences encoding P at codon 40 could be identified in available databases. The p60-MIT L40P:p80-CTD complex structure was solved using selenomethionine-labeled protein for obtaining experimental phases and refined to 2.4 Å resolution with two copies of a 1:1 p60-MIT L40P:p80-CTD complex in the asymmetric unit. The crystallographic R-factors for the final model and all available data between 43.3 and 2.4 Å resolution are 24.5/29.4 (Rwork/Rfree), which is rather high for this resolution range. The solvent content of the crystals is 53% and the protein molecules are arranged in layers with very few strong crystal contacts across the layers (Fig. S1). Three major disordered regions of the complex (both the whole N-terminal tag (40 residues) and one loop of p80 (residues 600–614 in chain A and 601–609 in chain C), and one loop of p60 (residues 37–48 in chain B and 37–45 in chain D)) are located at the contact planes between the individual layers in the crystal and likely explain the observed high crystallographic R-factors. A representative section of the electron density map covering both the p80 H498-T520 and the p60 Q57-F76 regions is shown in Fig. S2.

To estimate an apparent dissociation constant, K_d_, of the p60-MIT:p80-CTD complex and to confirm its 1:1 stoichiometry in solution we performed sedimentation velocity analytical ultracentrifugation (SV-AUC) experiments. In the 300 nM to 50 μM concentration range, the c(S) distribution showed a single peak with a s_w_ value of 2.4 S, which corresponds to a S_max_/S value of 1.5 (Fig. 1B). This result is consistent with p60-MIT and p80-CTD forming a tight, moderately elongated, heterodimeric complex in solution with an estimated K_d_ value in the low nano- or pico-molar range.

**Figure 2:**
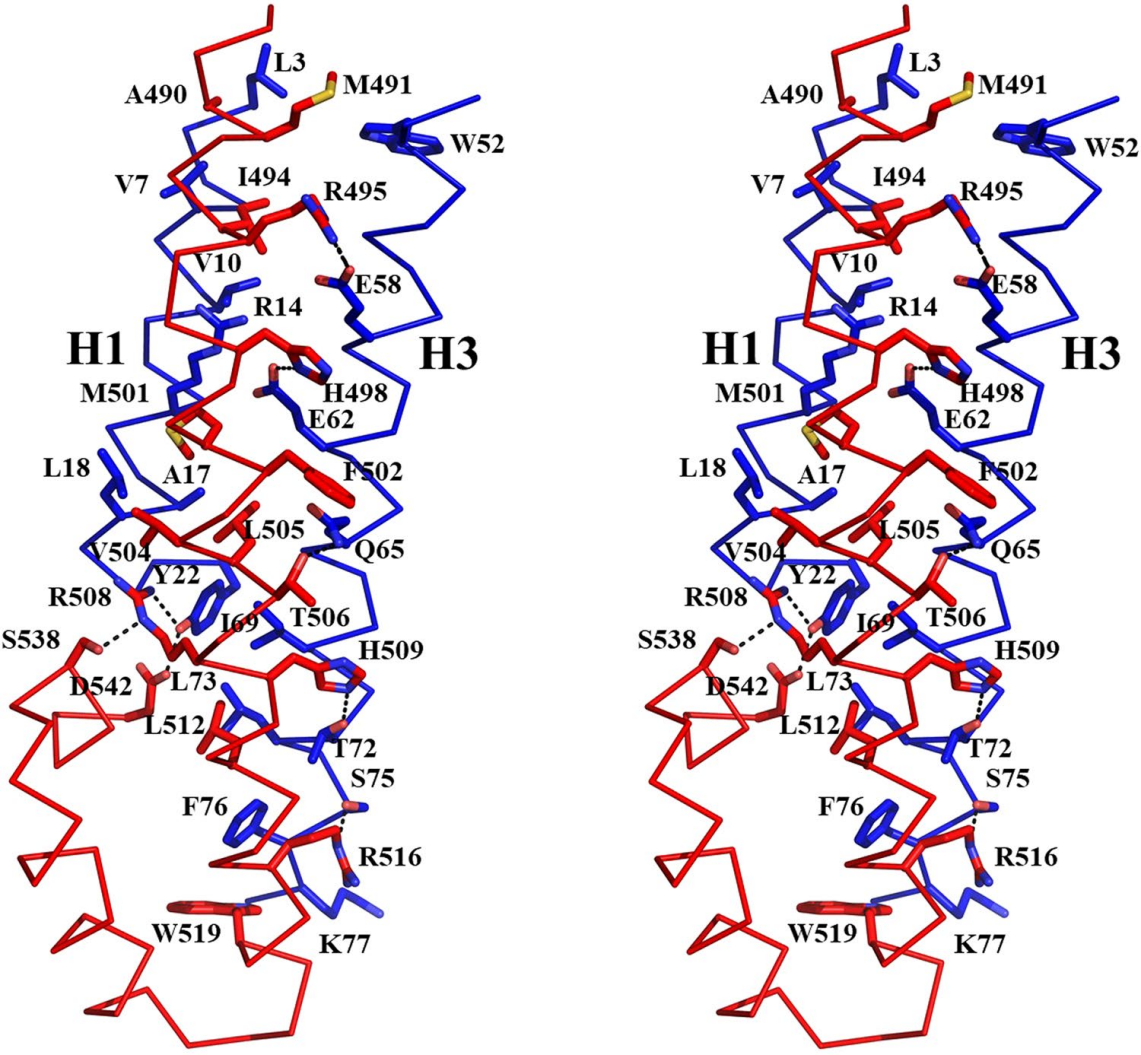
Zoom-in stereo view of a ribbon representation of the interaction interface between p60-MIT and p80-CTD with important residues highlighted in stick representation.

p60-MIT forms an antiparallel three-helix bundle that is characteristic of MIT domains. The p80-CTD domain consists of seven helices that adopt an extended helix-turn-helix fold. The geometry of the p60-MIT three-helix bundle creates shallow grooves between helices H1 and H2 and helices H2 and H3, and a deeper groove between helices H1 and H3. The 50 Å-long N-terminal helix of p80-CTD, comprising residues 487 to 521, is oriented in a parallel manner to helices H1 and H2 of p60-MIT. It fills the deeper groove formed between helices H1 and H3 of p60-MIT. The total surface area buried upon complex formation is 2774 Å^2^. Interactions between subunits are mediated by hydrophobic residues of p60-MIT and p80-CTD. p60-MIT residues L3, V7, V10, A17 and L18 of helix H1 and W52, V55, I69, L73 and F76 of helix H3 are the major hydrophobic residues in contact with A490, M491, I494, M501, F502, V504, L505, L512 and W519 of the N-terminal helix of p80-CTD. In addition, backbone and side chain hydrogen bonds are formed between residues R14, A17, L18, Y22, E58, E62, Q65, Y72, S75 and K77 of the p60-MIT helices H1 and H3 and I494, R495, H498, T506, R508, H509, R516, W519, S538 and D542 of the N-terminal helix of p80-CTD (Fig. 2). The residues forming the p60-MIT:p80-CTD interface are highly similar among katanin subunit homologs, suggesting that these interactions are conserved (data not shown).

**Figure 3:**
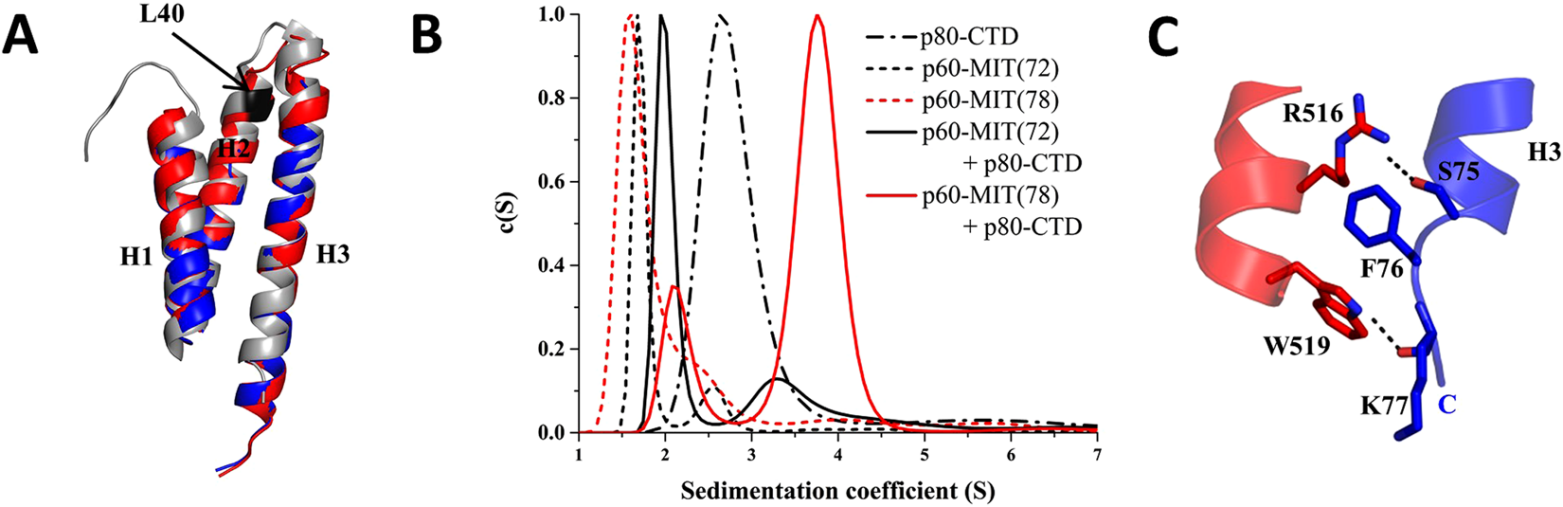
Importance of the C terminus of the p60-MIT domain for the p60-MIT:p80-CTD complex formation. (**A**) Superimposition of p60-MIT of the p60-MIT L40P:p80-CTD complex structure (in blue) with the p60-MIT solution NMR structure (PDB ID 2RPA, in grey), and the p60-MIT domain from the katanin:ASPM complex (PDB ID 5LB7, in red). The L40P mutation of p60-MIT that resulted in crystals suitable for structure determination, is highlighted in black. (**B**) c(S) distribution showing that the minimal version of p60-MIT (aa 1–72 of mouse p60) used to determine its NMR structure does not bind to p80-CTD. For technical reasons, these measurements were carried out with both proteins fused to thioredoxin. All proteins were used at a concentration of 20 µM. (**C**) Cartoon representation showing the interactions within the C-terminal part of the third helix of p60 that is crucial for p60-MIT:p80-CTD complex formation. Blue, p60-MIT; red, p80-CTD; black, hydrogen bonds. Interacting residues are highlighted as sticks.

**Figure 4:**
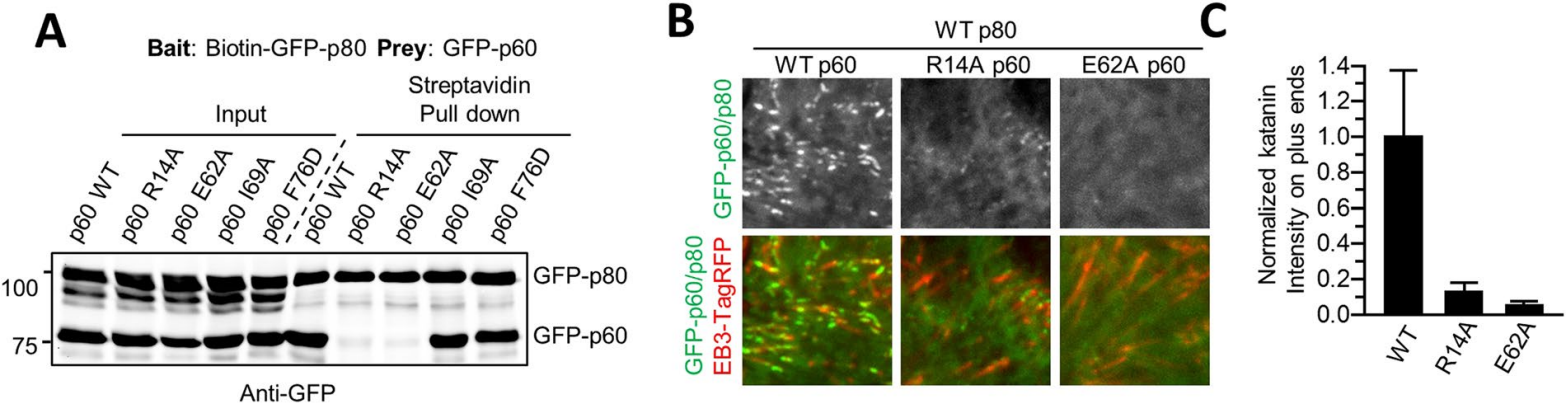
Significance of p60-MIT:p80-CTD interface residues for katanin heterodimerization and function. (**A**) Streptavidin pull-down assay with lysates of HEK293T cells co-transfected with indicated GFP-p80 and GFP-p60 mutants and biotin ligase BirA. (**B**) Maximum projection of 10 s time lapse movies of indicated GFP-p60/p80 mutants co-expressed with EB3-TagRFP (a marker of MT plus ends) in HeLa cells. Scale bar, 2 μm. (**C**) Quantification of p60/p80 intensity on MT plus ends. The intensity is normalized to the average intensity of the wild-type protein. Data represent mean ± SD. N = 10 cells per condition.

The overall structure of the p60-MIT L40P subunit complexed to p80-CTD is identical to the solution NMR structure of p60-MIT^14^ and to p60-MIT from the katanin:ASPM complex^12^. Superimposition showed that the L40P mutation disturbed the last two turns of helix H2 but has no impact on the overall architecture of the p60-MIT domain (Fig. 3A). The solution NMR structure of p60-MIT comprises only amino acids 1–72. Surprisingly, as shown by SV-AUC experiments (Fig. 3B), this domain variant does not bind to p80-CTD, suggesting that residues 73 to 78 might be important for the stabilization of the N-terminal helix of p80 (Fig. 3C). Notably, a single residue mutation within this amino-acid segment of p60-MIT (F76D) did not have a significant effect on the interaction (Fig. 4A), indicating the importance of the hydrogen bonds formed between the amino group of R516 of p80-CTD and the hydroxyl group of S75 of p60-MIT, and between the side chains of Y519 of p80-CTD and of K77 of p60-MIT for p60:p80 complex formation.

As there is no (or only a very weak) interaction between the shorter version of p60-MIT (residues 1–72) and p80-CTD, it is not possible to co-express and co-purify the truncated complex like the wild-type p60-MIT:p80-CTD complex. Because we did not succeed in producing p80-CTD alone, we fused p80-CTD to the C-terminus of N-terminally 6xHis-tagged *E. coli* thioredoxin A. This thioredoxin-p80-CTD fusion allowed us to obtain the protein in reasonable amounts and good quality. As can be seen from the crystal structure, a plausible explanation for the difficulties in expressing p80-CTD is that the hydrophobic part of the p80 domain’s N-terminal helix is strongly exposed when not complexed to p60-MIT, which is likely to result in a destabilization of the domain.

To provide further insight into the significance of single residues for the stability of the heterodimer, we mutated several conserved residues at the interface between p60-MIT and p80-CTD. Towards this end, a series of hydrophobic and two charged residues were selected based on the X-ray structure. Mutation of single hydrophobic residues (p60: V10A, L18A, I69A, F76A, F76D; p80: I494A, M501A, V504A, L505A, L512A) did not have a significant effect on the interaction in pull-down experiments (Fig. 4A and data not shown). However, we found that substitution of R14 or E62 to A greatly diminished the interaction between p60-MIT and p80-CTD (Fig. 4A). As seen in the crystal structure, the side chains of R14 and E62 of p60-MIT form hydrogen bonds to the backbone of I494 and the side chain of H498 of p80-CTD, respectively. Although the interaction surface between p60-MIT and p80-CTD is rather large and contains many hydrophobic contacts, this finding indicates that conserved charged residues at the interface are more critical than single hydrophobic residues for katanin p60:p80 complex formation.

Previous work has shown that the p80 subunit promotes katanin function by participating in its targeting to MTs^13^. We recently reported that when overexpressed in cells, the p60:p80 complex behaves as an unusual MT-associated protein that decorates growing MT tips and causes their bending and breakage^12^. This katanin behavior critically depends on the p80 C terminus, but not on the WD40 domain of p80^12^. MT end decoration thus provides a sensitive and specific assay for the formation of the complex between p60-MIT and p80-CTD. We overexpressed p80 together with the p60 R14A and E62A mutants in HeLa cells and examined their ability to track MT plus ends by using End Binding protein (EB3) labelled with TagRFP as a growing MT end marker (Fig. 4B,C). In agreement with the pull-down experiments, these mutants completely failed to localize to growing MT ends, demonstrating that when p60-MIT:p80-CTD complex formation is disrupted, p80 can no longer promote the recruitment of p60 to MTs. This finding therefore highlights the importance of p60:p80 complex formation for katanin function.

Three of the p80 katanin mutations, S538L, L543R and G581D that were recently identified in microcephaly patients are found within the p80-CTD domain^5^. The effect of these mutations can be rationalized on the basis of the crystal structure of the heterodimeric p60-MIT L40P:p80-CTD complex. L543 and G581 are located within the hydrophobic core of p80-CTD and their mutation by charged residues is expected to compromise the overall stability of the fold. S538 is located at the beginning of helix H3 of p80-CTD. Its hydroxyl group forms a intradomain hydrogen bond with R508. Therefore, its mutation to L is also predicted to destabilize the p80-CTD domain, but is not expected to affect the interaction with p60-MIT (Fig. 1A).

Taken together, the crystal structure of the p60-MIT:p80-CTD complex revealed a heterodimeric helical fold that is stabilized by a combination of hydrophobic and hydrogen bond interactions. Mutagenesis of single conserved residues at the interaction interface and functional analyses of the mutants in cells revealed the importance of charged residues for katanin p60:p80 complex formation.

### Comparison of p60-MIT with other AAA+ ATPases

MIT domains are present in various proteins involved in endosomal trafficking, MT binding or autophagy such as vacuolar sorting protein VPS4, katanin p60, spastin, spartin, sorting nexin SNX15 or autophagy related protein ATG1^14–17^. Although they are difficult to identify on the basis of sequence similarity, their three-dimensional structures are highly similar to each other. They all form an asymmetric three-helix bundle. Despite the apparent simplicity of the MIT domain structures, their ability to bind the MIT-interacting motif (MIM) of their binding partners is remarkably versatile. Several distinct binding modes have been identified for different pairs of MIT domains and MIM peptide motifs. To date, MIMs have been described to recognize the interface formed either between helices H1 and H3 or helices H2 and H3 of MIT domains^3,18^.

**Figure 5:**
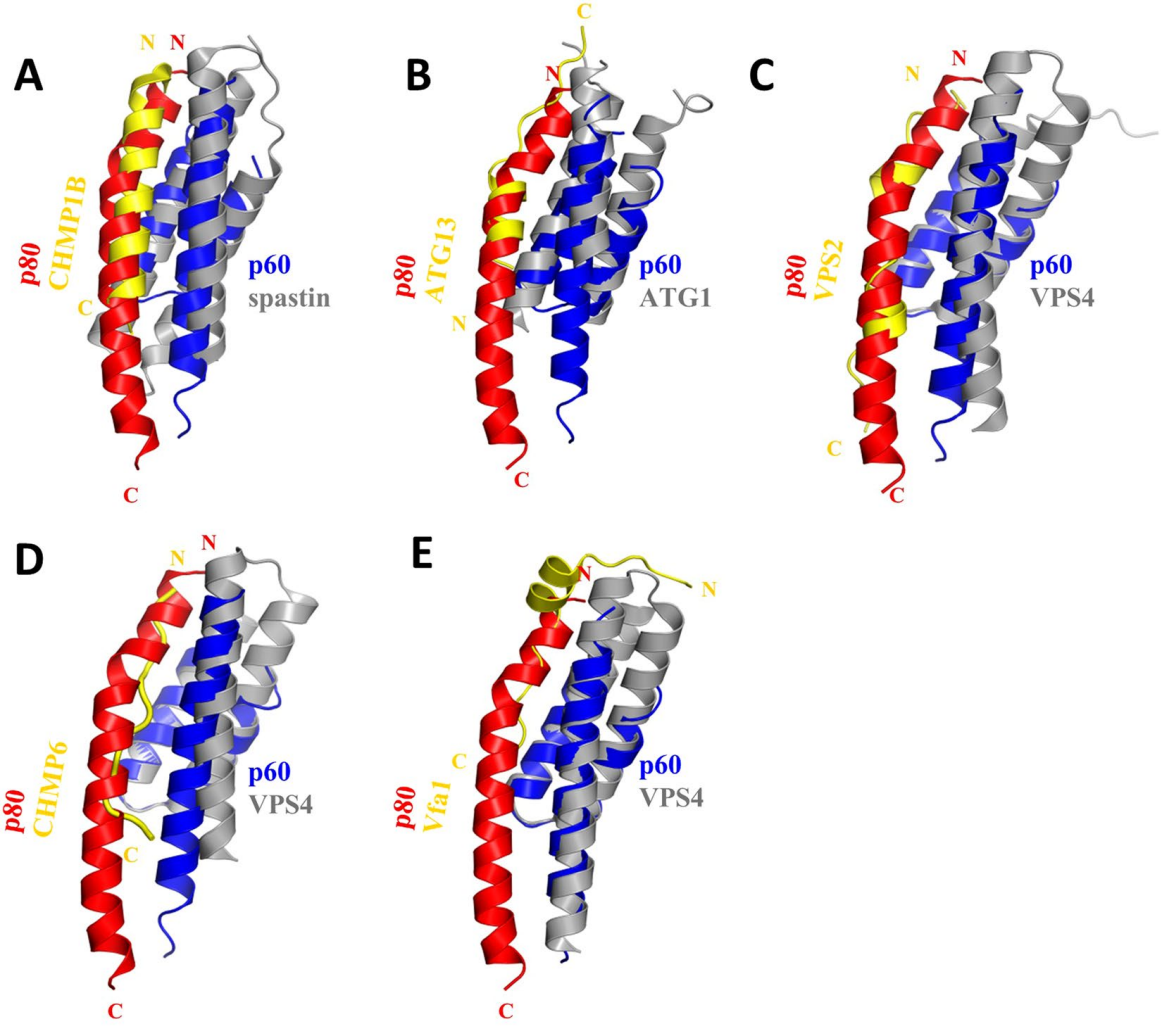
Comparison of katanin p60-MIT interactions with those of other AAA+ ATPses. (**A**–**E**) The structures of different MIT domains are highly similar to each other and they all form an asymmetric three-helix bundle. Superimposition of available complex structures demonstrates an overlap of the binding motifs, although the molecular contacts between these complexes are in most cases not conserved. (**A**) Spastin-CHMP1B structure (PDB ID 3EAB), (**B)** ATG1-ATG13 structure (PDB ID 4P1N), (**C**) Vps4-Vps2 structure (PDB ID 2V6X), (**D**) Vps4-CHMP6 structure (PDB ID 2K3W), (**E**) VPS4-Vfa1 structure (PDB ID 5FVK).

In katanin, the N-terminal helix of p80-CTD binds into the groove formed by helices H1 and H3 of p60-MIT. This mode of interaction is similar to those described for the spastin-CHMP1B, ATG1-ATG13, Vps4-Vps2, Vps4-Vfa1 or the VPS4-CHMP6 complex^15,16,19–21^. However, the sequence of CHMP6 corresponding to the N terminus of p80 adopts an unstructured, rather than the helical conformation seen in spastin and p80-CTD. The corresponding segments of Vps2 and Vfa1 adopt α-helical structures combined with unstructured parts. Superimposition of available complex structures demonstrates an overlap of the binding motifs (Fig. 5A–E), although the molecular contacts between these complexes are in most cases not conserved. Moreover, the N-terminal helix of p80-CTD is longer than other MIMs and undergoes additional interactions with the C terminus of helix H3 of the p60–MIT domain that are not observed in other MIT-MIM complexes (Figs 3C and 5A–E).

The interaction between p60-MIT and p80-CTD complex is very strong with an estimated K_d_ in the low nanomolar to picomolar range. This is a significantly tighter binding than previously described for most MIT-MIM interactions. With the exception of the CHMP3-AMSH interaction that was reported to have a K_d_ of about 60 nM^22^, the measured affinities are usually in the range of 2 to 100 µM. The tight interaction between p60-MIT and p80-CTD can be explained by a large total surface area of 2774Å^2^ buried upon complex formation as compared to, for example, 1238 Å^2^ of the Vps4 MIT-Vps2 complex (K_d_ 30 μM)^16^, 1946 Å^2^ of the spastin-CHMP1B complex (K_d_ 12 μM)^19^ or only 543 Å^2^ for the VPS4B-IST1 (K_d_ 11 μM) complex^23^. In all these complexes, hydrophobic interactions play a crucial role. In contrast, the CHMP3-AMSH complex structure is mainly stabilized by hydrogen bonds and salt bridges while hydrophobic contacts seem to play only a minor role. The 60 nM K_d_ observed for this complex with a total surface area buried upon complex formation of 1650 Å^2^ is to date the strongest K_d_ reported for a MIM-MIT interaction^22^. Notably, hydrogen bonds also critically contribute to the high-affinity association of p60-MIT with p80-CTD, although the interaction interface between subunits is dominated by a large network of hydrophobic interactions (Fig. 2).

Taken together, the mode of interaction seen in the crystal structure of the katanin p60-MIT:p80-CTD heterodimer is similar to those observed for other complexes formed between MIT domains and MIM motifs. The low K_d_ of the interaction can be explained by the large buried surface area formed by the katanin heterodimer.

## Conclusion

The goal of this study was to explore the molecular details of the interaction between katanin subunits mediated by p60-MIT and p80-CTD using biophysical, structural and functional methods. We found that p60-MIT and p80-CTD form a heterodimer that is significantly stronger than most of the other known MIT-MIM interactions. Our findings provide new insights into katanin structure, reveal conserved charged residues that are important for p60-MIT:p80-CTD complex formation and highlight the complexity of protein recognition by MIT domains. An important next step will be to elucidate the precise mechanistic basis of MT severing by katanin, knowledge that will help to understand how this fascinating enzyme drives a wide array of essential biological processes.

## Methods

### DNA constructs and protein production

The p60-MIT:p80-CTD complex was cloned, co-expressed and purified as described previously^12^. Shortly, p60-MIT:6xHis-p80CTD was expressed in pET28a (Novagen) using BL21(DE3) cells. The recombinant protein was purified by IMAC on a 5 ml HisTrap FF Crude column (GE Healthcare) according to the manufacturer’s instructions, followed by a size-exclusion chromatography step on a Superdex 75 column in 20 mM HEPES, pH 7.4, supplemented with 150 mM NaCl. cDNAs encoding mouse p60-MIT (residues 1–70 and 1–78) and mouse p80-CTD (residues 481–658) were cloned into a modified version of the pET-15b vector, pHisTrx2, which includes E. coli thioredoxin A as a fusion protein after the His_6_ tag. All mutants were generated either using the QuikChange method or the Gibson assembly reaction.

### Analytical ultracentrifugation (AUC)

Sedimentation velocity (SV) experiments were performed at 20 °C and 42,000 rpm in a Beckman Coulter ProteomeLab XL-I analytical ultracentrifuge using standard protocols^24,25^. All measurements were conducted in 20 mM HEPES, pH 7.4, supplemented with 150 mM NaCl and 2 mM 2-mercaptoethanol and data were acquired using absorbance (280 and 250 nm) or interference optical systems. Protein partial specific volumes, solution density and viscosity were calculated in SEDNTERP (http://sednterp.unh.edu/). Data analysis was performed with the SEDFIT software package using a sedimentation coefficient distribution model c(S)^26^. Scan file time stamps were corrected^27^ and good fits were obtained with r.m.s.d. values corresponding to typical instrument noise values. Sedimentation coefficients were corrected to standard conditions, s_20,w_. The shape and oligomerization state of the proteins were determined on the basis of the Smax/S ratio. Smax was calculated by the following equation: Smax = 0.00361 × M2/3, where M is the molecular weight of the protein^28^.

### Crystallization and X-ray structure determination

The p60-MIT L40P:p80-CTD complex in 20 mM HEPES, pH 7.5, supplemented with 150 mM NaCl, was concentrated to 20 mg/ml prior to crystallization. Initial crystals of the p60-MIT L40P:p80-CTD complex appeared within a few hours at 20 °C using the sitting drop method in 20% PEG 3350, 0.1 M BisTris propane (pH 6.5), 0.2 M sodium bromide. These crystals were used for one round of microseed matrix screening. Crystals suitable for structure determination were obtained at 20 °C in sitting drops composed of a 1:1 mixture of the protein solution and a well solution consisting of 27% PEG 3350, 0.1 M BisTris propane (6.0), 0.2 M MgCl2 and 10% of the seed stock. Crystals of the selenomethionine-labeled p60-MIT L40P:p80-CTD complex were obtained under the same conditions.

Native and selenomethionine MAD data sets of p60-MIT L40P:p80-CTD were acquired at the beamline X06DA of the Swiss Light Source (Paul Scherrer Institute, Villigen, Switzerland) to a resolution of 2.4 Å and 3 Å, respectively. The acquired datasets were reduced, scaled and merged using XDS, XSCALE and XDSCONV^29,30^. The p60-MIT L40P:p80-CTD structure was solved in the C2 space group using AUTOSHARP^31^. The initial model building was done via AUTOBUILD and was taken as molecular replacement search model for phasing the native dataset by PHASER^32^. Data and refinement statistics are reported in Data Table S1. The structure was deposited in the PDBe databank under accession code 5NBT. Molecular graphics and analyses were performed with PyMol (The PyMOL Molecular Graphics System, Version 1.5.0.5. Schrödinger, LLC).

### Pull-down and MT end tracking assays

HeLa and HEK293T cells were cultured in DMEM/F10 (1:1) supplemented with 10% FBS and 5 U/ml penicillin and 50 μg/ml streptomycin. HeLa and HEK293T cell lines used here were not found in the database of commonly misidentified cell lines maintained by ICLAC and NCBI BioSample, were not authenticated and were negative for mycoplasma contamination. FuGENE6 (Promega) was used to transfect plasmids in HeLa cells; polyethylenimine (PEI, Polysciences) was used to transfect HEK293T cells for pull down experiments. Streptavidin pull-down assays with lysates of HEK293T cells co-transfected with GFP-p80 and GFP-p60 variants and biotin ligase BirA were performed using streptavidin beads (Invitrogen) as described previously^33^.

TIRF microscopy performed on an inverted research microscope Nikon Eclipse Ti-E (Nikon) with the perfect focus system (PFS) (Nikon), equipped with the Nikon CFI Apo TIRF 100 × 1.49 N.A. oil objective (Nikon), Photometrics Evolve 512 EMCCD (Roper Scientific) camera controlled with the MetaMorph 7.7 software (Molecular Devices). Images were projected onto the chip of Evolve 512 camera with intermediate lens 2.5X (Nikon C mount adapter 2.5X). The final magnification was 0.063 μm/pixel. To keep cells at 37 °C or *in vitro* samples at 30 °C we used stage top incubator INUBG2E-ZILCS (Tokai Hit).

For excitation we used 491 nm 100 mW Stradus (Vortran) and 561 nm 100 mW Jive (Cobolt). We used ET-GFP 49002 filter set (Chroma) for imaging of proteins tagged with GFP, ET-mCherry 49008 filter set (Chroma) for imaging EB3-TagRFP. For simultaneous imaging of green and red fluorescence, we used the triple-band TIRF polychroic ZT405/488/561rpc (Chroma) and the triple-band laser emission filter ZET405/488/561 m (Chroma), mounted in the metal cube (Chroma, 91032) together with Optosplit III beamsplitter (Cairn Research Ltd, UK) equipped with a double emission filter cube configured with ET525/50 m, ET630/75 m and T585LPXR (Chroma).

### Image processing

Images were prepared for publication using ImageJ and Adobe Photoshop. All images were modified by adjustments of levels and contrast. Quantifications were performed in ImageJ.

### Data availability

All data generated or analysed during this study are included in this published article. The structure of p60-MIT L40P:p80-CTD complex was deposited in the PDBe databank under accession code 5NBT.

## Electronic supplementary material

Supplementary Information
