# Supplementary material for: Structural basis of katanin p60:p80 complex formation
Source: Sci Rep. 2017 Nov 2;7:14893. doi: 10.1038/s41598-017-14194-2 (PMC5668312; doi:10.1038/s41598-017-14194-2)

Structural basis of katanin p60:p80 complex formation

Lenka Rezabkova[1]*, Kai Jiang[2]*, Guido Capitani[1], Andrea E. Prota[1], Anna Akhmanova[2], Michel O. Steinmetz[1,3#] and Richard A. Kammerer[1#]

* These authors contributed equally

[#] Corresponding authors: richard.kammerer@psi.ch, michel.steinmetz@psi.ch

[1]Laboratory of Biomolecular Research, Division of Biology and Chemistry, Paul Scherrer Institut, CH-5232 Villigen PSI, Switzerland

[2]Cell Biology, Department of Biology, Faculty of Science, Utrecht University, Padualaan 8, 3584 CH Utrecht, The Netherlands.

[3]Biozentrum, University of Basel, CH-4056 Basel, Switzerland

Table S1 Crystallographic data collection and refinement statistics.

Fig. S1 Packing of p60-MIT L40P:p80-CTD crystals.

Crystal packing in two orientations highlighting the contact planes formed by the disordered regions of the molecules in the crystal. The individual chains of the two copies of the p60-MIT L40P:p80-CTD complex are in green, cyan, magenta and yellow ribbon representation. The terminal residues of the disordered regions are highlighted as spheres in the corresponding chain colours.

Fig. S2 Representative electron density map of the p60-MIT L40P:p80-CTD structure

Representative section of the electron density map covering both the p80 H498-T520 and the p60 Q57-F76 regions. The SigmaA - weighted 2mFo - DFc (dark blue, contoured at +1.0σ) and mFo - DFc (light green, +3.0σ; red, -3.0σ) electron density maps are shown. The p80-CTD and p60-MIT L40P molecules are in red and dark blue stick representation. Oxygen and nitrogen atoms are coloured in red and blue, respectively.

Table S1

	p60-MIT L40P:p80-CTD
**Data collection**	
Space group	C2
Cell dimensions	
a, b, c (Å)	146.1, 37.8, 103.1
α, β, γ (°)	90, 93.4, 90
Resolution (Å)[a]	43.3-2.4 (2.6-2.4)
No. reflections	22520 (4734)
R_{meas} (%)	17.8 (151.6)
$CC_{1/2}$	99.8 (65.9)
I / σI	8.9 (1.5)
Completeness (%)	99.9 (100.0)
Redundancy	6.7 (7.0)
**Refinement**	
Resolution (Å)	43.3 – 2.4
No. unique reflections	22494
R_{work} / R_{free} (%)	24.5 / 29.4
Average B-factors (Å2)	
Complex	64.5
Solvent	56.7
Wilson B-factor	48.0
Root mean square deviation from ideality	
Bond length (Å)	0.003
Bond angles (°)	0.582
Ramachandran statistics	
Favored regions (%)	98.8
Allowed regions (%)	1.2
Outliers (%)	0

[a]Highest-resolution shell is shown in parentheses.

Fig.S1

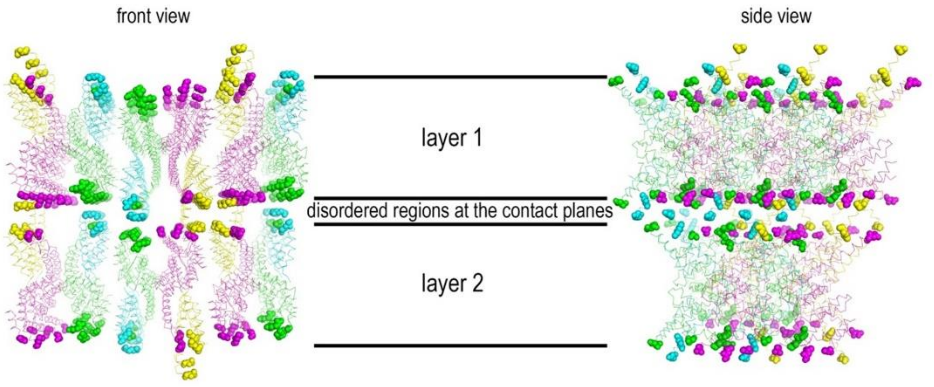
front view
side view
layer 1
disordered regions at the contact planes
layer 2

Fig.S2

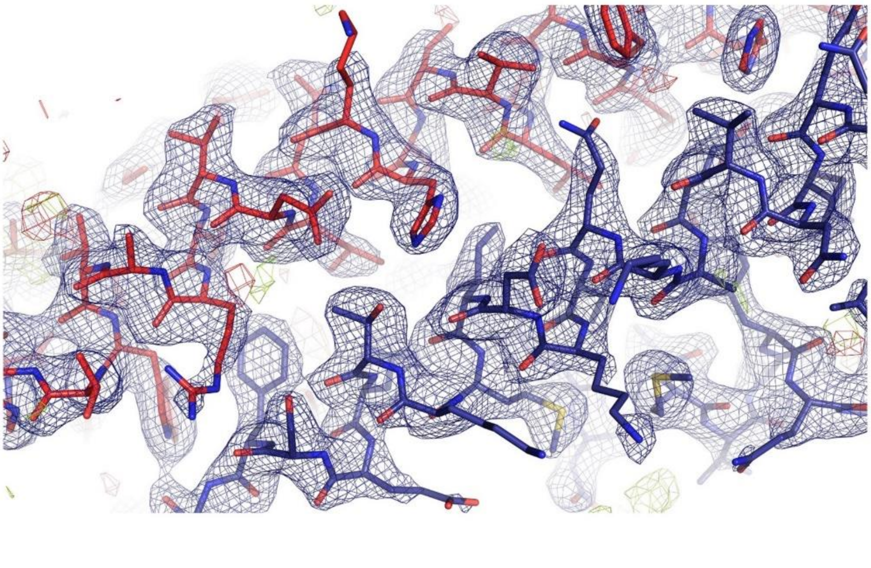

Supplement: Supplementary file 1 — Supplementary Information [file 41598_2017_14194_MOESM1_ESM.pdf]
